# Mad Itch

**Published:** 1890-07

**Authors:** S. Stewart

**Affiliations:** Council Bluffs, Iowa


### MAD ITCH.

By S. Stewart, M.D., D.V.M.

The following description of this disease is placed before the readers of the Journal, hoping that someone has discovered the etiology and pathology of this peculiar malady, and will inform his less fortunate brethren of his findings through the columns of this journal.

Correspondence with several well-known members of our profession elicits the fact that many others share the writer's lack of definite notions of this disease, and will gladly receive any light the fortunate possessor will shed upon this subject.

It has been called by many names, the most popular of which is "*mad itch*;" but, so far, no name has included in its meaning any definite intimation of the cause or special lesions of this disease.

In western Iowa and eastern Nebraska, and for aught the writer knows, in a much greater territory, this disease develops during, or directly following, severe Winter weather, usually affecting but a single herd in any given neighborhood. The general condition of the cattle attacked nor their environment seems to predispose to or influence the malady. So far as the writer's observation goes, females only are subject to this disease, as can be seen from the following notes of cases :

December, 1886, Williams & Powell, of Guthrie Centre, Ia., had on their farm about 110 head, pure bred and grade, Hereford cattle. There were seven or eight pure bred cows, five pure bred bulls, the remainder being grade calves, yearlings and cows. Part of the calves were males. These cattle were sheltered in the basement of a well-ventilated barn, and fed on clean, bright prairie hay, with one bundle each of sheaf oats for grain. The oats and straw were very bright, free from rust or fungi, and had the sweet odor natural to this grain when well cured. These stables were cleaned daily, the droppings being carted out on the fields. The grade cattle were watered from a small stream, distant about forty rods from the barn ; the pure bred cattle were supplied from a well. These cattle were turned out in yards for several hours daily, and all seemed to be in a thriving condition.

On Thursday evening, December 16th, 1886, Bessie, a two-year-old grade heifer, was observed to switch her tail peculiarly, then soon to lick and bite or rub her nates and stamp her hind feet. The muscles of the gluteal and crural regions jerked in a choreic manner. This condition was soon intensified so that she would frequently lie down, soon to rise again. or sit upon her haunches and slide the parts backward and forward upon the ground ; would rub the buttocks against the enclosure with sufficient violence to abrade, and even lacerate the parts rubbed ; would stamp the hind leg as though covered with flies which she was determined to dislodge. Posterior muscular paralysis soon followed, and she could not rise. The irritating sensations continued so annoying in her feet and legs that she would roll from side to side, over the sternum, and whip the uppermost limb on the ground, also lick and bite it until it would bleed. Next came contraction of the levator muscles of the head and neck, which brought the head nearly over to the dorsal region. Death ended her sufferings in thirteen hours. The appetite was lost, but she had power to chew and swallow food, and did so at times, apparently through sheer agony. The power to regurgitate was lost from the beginning of symptoms ; bowels constipated, urine sufficient, thirst limited, respiration hurried, temperature and pulse not taken. Post-mortem examination by the owners, who were practical butchers and close observers, showed mottled lungs, distended bladder, maniplies rather dry, but nothing to attract special attention.

Saturday morning, December 18th, 1886, Daisy, a three-year-old grade, located in the opposite end of the stable from Bessie, was seen to present the initial symptoms of the preceding case. Mr. Ray, a practitioner of Des Moines, Ia., was consulted by telephone, who pronounced it inflammation of the womb, and prescribed for that disease. This cow repeated the same line of symptoms as Bessie, and died thirty hours after the first symptoms were seen.

Sunday morning found Jessie and Princess, two-year-old grades, showing the first evidences of this disease, although quite well the evening preceding, when all the cattle were carefully inspected. Thirty and thirty-five hours, respectively, terminated their lives in the same manner as before described.

On this Sunday morning, December 19th, the pure-bred cows were driven to another farm, one and one-half miles distant. At eight P. M. of the same day, Countess, a six-year-old pure-bred cow, commenced to switch her tail and have choreic jerks of the gluteal muscles. She was immediately driven back to the home farm. Mr. Ray, of Des Moines, had now arrived, and diagnosed dry murrain, prescribed cathartic doses of salts for the whole herd of cattle, as all were, he thought, slightly constipated. The cathartic acted freely on the sick and well alike. The sick were not relieved. The same evening Nancy, a three-old grade, showed the first symptoms. Copious drenchings with salines produced liquid stools, yet the course of the disease was not stayed. The progress of symptoms was not so rapid in these cases.

When first examined by the writer, on the evening of December 21st, the cow Nancy was unable to rise, but rolled herself from side to side over the sternum to bite and lick her hind legs and feet. Found her temperature 105°, respirations 40, pulse imperceptible at the jaw. Moist bronchial râles; good resonance over the lungs. Gave large doses of fluid extract of belladonna and fluid extract of gelsemium at short intervals during twenty-four hours without any apparent effect upon the disease. Opisthotonos developed during the 23d, and by night the head was drawn well over to the back. She now lay flat on her side and moaned with each breath. She was bled to death for post-mortem examination.

At nine P. M., December 22d, Juniper, a six-year-old pure-bred cow, which had been away from the home farm three days, was attacked with the fatal malady, and was immediately driven to the home farm. I saw her at noon of the 23d. Found her temperature 105° ; respirations 50 ; pulse 80, small soft ; bowels active ; urine abundant ; restless, frequently getting up and down, violently rubbing and biting her hind legs and buttocks. Gave of fluid extracts of belladonna and gelsemium, each one ounce, at twelve M., one and three P. M., without modifying the symptoms. At five P. M. Prof. M. Stalker, of Ames, Ia., examined the cow, and advised the use of fluid extract of ergot and potassium bromide, which was given in one-half-ounce doses of each, every two hours, all night and the following A. M., without producing any apparent effect. Hypodermic injection of these drugs was then resorted to, but no modification of the course of the disease was obtained. She lived ninety-six hours.

Post-mortem examination, made several hours after the death of Countess, showed the lungs mottled dark and light-red ; some lobules collapsed ; heart normal, containing a soft clot ; pericardium and pleura, also vessels of thorax, normal ; rumen, recticulum omasum were full, contents dry ; abomasum and small intestines empty ; liver very large ; spleen small and soft ; kidneys normal ; bladder full of dark frothy urine ; womb normal and contained a healthy looking fœtus ; spinal dura mater normal in color ; arterial congestion of the pia mater ; the cord of firm consistency ; choroid plexus a bright-red color and surrounded by considerable fluid ; other parts of brain quite normal.

In Jessie and Princess the viscera of the thorax and abdomen were similar to those of Countess, but the dura mater of the spinal cord in the dorsal and lumbar portions was intensely red, and the cord in these portions very soft, like cerebral substance.

Nancy, the cow destroyed for the post-mortem study, was carefully examined by Prof. M. Stalker and myself. The genital and urinary organs were removed and inspected, but nothing abnormal was found. The digestive organs presented nothing unusual ; liver rather large and pale in color ; the spleen soft and small ; lungs congested ; heart rather flabby. The dura mater of the brain and cord was normal in color. An excess of fluid surrounded the cord, with a very limited quantity in the cerebral ventricles. There was engorgement of the pia mater, choroid plexus and tenia semicircularis. The cerebrum was quite firm, the cerebellum very soft, while the spinal cord was of firm consistency throughout its entire length.

No more cases occurred in this herd of cattle at that date, nor since.

In March, 1886, Mr. A. Fulton, who lives in Cass Co., had twenty yearlings, ten each steers and heifers. They were provided with an open shed for shelter ; ran loose in a feed yard ; ate shelled corn from large boxes and well-cured timothy hay from a rack ; drank deep well water from a tank. Of this bunch of young cattle four heifers sickened and died. They presented the same train of symptoms as the Guthrie Centre herd just described. No cases like them were ever known in that vicinity before nor since.

About February 1st, 1890, George Smith, living near Carson, Pottawattamie Co., Ia., lost by sickness six cows and heifers which he was feeding for beef in an unprotected feed yard. The remainder of the bunch, consisting of steers, cows and heifers, escaped the disease. The description of how these cattle acted when sick, as given by Mr. Smith, was almost identical with the preceding discription. A butcher made a post-mortem examination and was astonished to find "nothing wrong with her insides." These cattle were fed on shelled corn and millet hay; watered from a tank supplied from a deep well. One hundred or more young steers and heifers were yarded in an adjacent enclosure, fed from the same millet stack, watered from the same tank, but secured their corn from a large corn-stalk field. None of these young cattle were sick.

*Council Bluffs, Iowa.*

